# Clinical Trials of Senolytics in Alzheimer’s Disease Treatment and Prevention

**DOI:** 10.1093/geroni/igaf122.1102

**Published:** 2025-12-31

**Authors:** Miranda Orr

**Affiliations:** Washington University in St. Louis, St. Louis, Missouri, United States

## Abstract

Senolytics, agents that selectively eliminate senescent cells, have emerged as a promising geroscience-guided strategy for preventing and treating Alzheimer’s disease (AD). In preclinical models, senescent cells accumulate in the aging brain and contribute to neuroinflammation, tau and amyloid pathology, and cognitive decline. Early-phase human studies have begun to evaluate the feasibility, safety, and biological activity of senolytics in both symptomatic and at-risk older adults. Two open-label Phase 1 trials, SToMP-AD and STAMINA, evaluated 12 weeks of intermittent oral dasatinib plus quercetin (D+Q) in older adults. SToMP enrolled 5 participants with symptomatic AD (mean age 72 ± 4 years, 60% female) and assessed central nervous system (CNS) penetrance, safety, CSF and plasma biomarkers, and brain imaging. STAMINA enrolled 12 older adults with mild cognitive impairment (MCI) and slow gait speed (mean age 77 ± 8 years, 58% female) and evaluated changes in cognition, physical function, and blood-based biomarkers. Plasma levels of D and Q increased in all SToMP participants, and D was detected in CSF in 80% (range 0.281–0.536 ng/mL), confirming CNS exposure; Q was not detected in CSF. In SToMP, plasma inflammatory markers—including factors of senescence associated secretory phenotype (SASP)—decreased following treatment, a trend that was similarly observed in STAMINA. In SToMP, CSF levels of IL-6 and GFAP increased, while the CTRA gene expression profile decreased in four of five participants, suggesting downregulation of systemic immune stress. In STAMINA, reductions in plasma TNF-α significantly correlated with improvements in MoCA scores (r = −0.65, p = 0.02). These Phase 1 studies support the safety, feasibility, and biological activity of senolytic therapy in older adults with or at risk for AD. Plasma-based reductions in inflammation and SASP factors, CNS penetrance of dasatinib, and early signals of cognitive benefit offer a foundation for ongoing clinical development. A Phase 2 randomized controlled trial of D+Q in AD is now underway to further evaluate target engagement and clinical outcomes; interim findings will be presented. Together, these studies contribute to growing momentum in the field to develop senolytics as a novel disease-modifying approach targeting fundamental aging processes in AD.

